# Surgery

**Published:** 1906-01-06

**Authors:** 


### SURGERY.

**Surgery of Cleft Palate.**—Since Smith described his operation for cleft palate in the "Medico-Chirurgical Transactions" of 1868, that operation has been the classical one for this condition, and, as will be mentioned later, some authors still consider that it leaves nothing to be desired. It consists in paring the edges of the cleft and sewing them together after relieving tension by making lateral incisions through the tendons of the levator and tensor palati. But dissatisfied with the results of this operation, Davies Colley introduced, and Arbuthnot Lane has elaborated, a flap method of operation of a much more complicated nature. In this latter method the child's age should be about three months, as compared with the three-year age, which is that of choice in Smith's operation. The reason for choosing this early age for operating is that the teeth have not developed. A muco-periosteal flap is cut from one side of the cleft, and it includes the tissue covering the alveolar margin of the jaw. This flap is turned over and fixed between the bone and soft tissues on the opposite side of the cleft. The two great drawbacks to this operation are the severe shock sustained by a child of such tender years, and the large raw area left in the mouth to be covered by granulation. In 1901 Brophy,[1] a Chicago dentist, introduced a new method which differs fundamentally from either of the above, inasmuch as it approximates the bones and does not merely cover over the cleft between them. He operates at any age between ten days and three months, after which time the facial bones are too firmly ossified to allow of their being moved.

The stages in the operation are as follows: (1) The edges of the cleft are pared down to the cartilage of the maxilla. (2) Two or three stout wires are passed right across from the outer surface of one maxilla to that of the opposite, above the palatal processes. This generally has to be accomplished by passing silk loops from the outer side of each maxilla to the median cleft, and then threading one into the other; draw it right through both bones, and withdraw it in the reverse direction carrying the wire. (3) Lead plates are made to fit the convexity of the buccal surfaces of the bones and the ends of the wires threaded through holes in these plates, and the ends twisted together whilst the maxillæ are approximated by the pressure of the thumb and finger. (4) If the cleft is so wide that it cannot be closed by this manœuvre, the malar process may be divided by a scalpel, as it is chiefly cartilaginous in infants of three months. (5) The edges of the soft tissue bounding the cleft are united by interrupted sutures. (6) The premaxilla is pared on its inner surface and united to the anterior extremity of the maxillæ. Brophy states that, as the procedure is carried out at such a young age before the teeth have errupted, there is no deformity of the upper teeth produced in later life, but that these grow out at right angles to the replaced maxillæ. Also that there is little or no ultimate disparity in the size of the upper and lower jaw. The criticism of Brophy's operation in this country has been very varied, but the majority of surgeons who have had any experience of it admit that it makes a most valuable addition to our resources in this difficult class of case. Stokes [2] speaks very highly of the operation, and relates three successful cases of its application in infants of four weeks, six weeks, and three months. He prefers not to pare the edges of the cleft until after the bones have been approximated, thereby avoiding a good deal of hæmorrhage. On the other hand, Berry [3] condemns the method as being "violent" and "brutal," and states that although he has never performed it, he knows of eleven cases in which it was used, five of which ended fatally, and one other led to necrosis of the jaw! This author is equally scathing in speaking of the Davies Colley flap operation, and considers that the chief object of such operations is to exhibit the skill of the operator. He relates the results he himself has obtained by Thomas Smith's operation performed generally in the third year of life. Out of sixty-seven cases only three were failures, but in twenty-six one or more subsequent operations were necessary for closure of the defect in the hard palate, and five of the soft palate. Also he admits that "in no cases has speech after operation become absolutely normal, and that there are comparatively few in which I should call it very good." And it is only by operating at a much earlier age—*i.e.* within the first three or six months of life that defects of speech can be prevented, and as Smith's operation generally fails when done so early, Brophy's method cannot be dismissed without much more extensive trial. Owen [4] makes a most valuable suggestion for dealing with cases of cleft palate in which the sutured edges break down entirely after operation. He points out that this only too common accident is due to septic invasion from the mouth which cannot be prevented. The usual custom is to leave the case for some months before making an attempt to operate again. But that if instead of doing this the case be watched until the septic and sloughy edges have become clean, which takes only ten days or a fortnight, and they are then reunited without any further paring, union will readily take place, as the patient is temporarily immunised against septic infection and the healing surfaces are protected by granulations.

[1] Dental Cosmos, 1901. [2] Brit. Med. Jour., June 24, 1905. [3] Ibid., Oct. 7, 1905. [4] Ibid., June 24, 1905.

